# Lobectomy vs Segmentectomy in Stage I Non-Small Cell Lung Cancer With Lymphatic Vascular Invasion

**DOI:** 10.1016/j.atssr.2024.12.011

**Published:** 2025-01-07

**Authors:** Steven Stanley, Marco Braaten, Anjali Mishra, Ali Bin Abdul Jabbar, Muhammad Sohaib Asghar, Abubakar Tauseef, Peter Silberstein, Marcus Balters, Erin Gillaspie, Bradley DeVrieze

**Affiliations:** 1 Creighton University, School of Medicine, Omaha, Nebraska; 2 Department of Internal Medicine, Creighton University, School of Medicine, Omaha, Nebraska; 3 AdventHealth, Sebring, Florida; 4 Department of Nephrology, Mayo Clinic, Rochester, Minnesota; 5 Department of Hematology and Oncology, Creighton University School of Medicine, Omaha, Nebraska; 6 Department of Cardiothoracic Surgery, Creighton University, School of Medicine, Omaha Nebraska

## Abstract

**Background:**

Non-small cell lung cancer (NSCLC) constitutes 80% of new lung cancers and causes significant morbidity and mortality. Surgical interventions include lobectomy and segmentectomy. Lymphovascular invasion (LVI) correlates with reduced survival and increased recurrence; however, treatment recommendations are lacking. The aim of this study was to analyze survival and clinical characteristics of segmentectomy vs lobectomy in patients with stage I NSCLC with LVI.

**Methods:**

This retrospective cohort study, using the National Cancer Database from 2004 to 2020, identified patients with a diagnosis of NSCLC. Patients were propensity score matched by age, race, tumor size, and sex, divided into lobectomy and segmental resection groups. Statistical methods involved χ^2^, analysis of variance, Kaplan-Meier survival analysis, multinomial logistic regression, and the Cox proportional hazards model. Data were analyzed using SPSS software version 27.0 (IBM Corp), with a significance threshold of *P* <.05.

**Results:**

Of 8028 patients meeting inclusion criteria, 7612 (94.8%) underwent lobectomy and 416 (5.2%) underwent segmentectomy. After propensity score matching, there remained no significant differences in median survival between the segmentectomy group and The lobectomy group (75.2% [95% CI, 64.1%-86.3%] vs 76.1.8% [95% CI, 66.1%-86.0%], respectively; *P* = .922) Multivariate analysis of segmentectomy vs lobectomy showed no difference in hazard between the groups (Cox proportional model hazard ratio, 1.01; 95% CI, 0.837-1.22; *P* = .922) or odds of death (unadjusted logistic regression, .826; 95% CI, 0.300-0.421; *P* = .178).

**Conclusions:**

The main finding of this large, retrospective cohort study is that there was no significant difference in overall disease-free survival between patients who underwent segmental resection and patients who underwent lobectomy. After 1:1 propensity matching, there remained no significant difference between the 2 groups.

**In Short**

▪ After propensity score matching, there was no significant difference between segmentectomy and lobectomy in patients with LVI.
▪ Our analysis found that patients undergoing segmental resection were significantly older than those undergoing lobectomy, segmental resection was associated with a significantly shorter length of stay compared with lobectomy resections, and segmental resections were more likely to be performed at academic or research hospitals because of adherence to quality control measures.

Non-small cell lung cancer (NSCLC) is a heterogenous group of lung cancers comprising more than 80% of new lung cancer diagnoses and remains the leading cause of cancer-related deaths worldwide.^1^^,^^2^ Surgical resection is the mainstay of treatment for early-stage NSCLC. In 1995, the landmark Lung Cancer Study Group established lobectomy as the standard of surgical care for treatment of T1 N0 NSCLC.^3^ Segmental resection was recognized as an option for patients with impaired cardiopulmonary reserve. Recent large studies such as JCOG 0802 and CALGB 140503 found no significant difference in recurrence, disease free survival, and overall survival between lobectomy and segmental resection.^1^^,^^2^ Studies have demonstrated that lobectomy is less well tolerated and carries higher risk of postoperative complications and decreased lung function.^4^ These studies, in conjunction with other large meta-analyses, have challenged the superiority of lobectomy, thus inspiring further investigation of segmental resection as a treatment for early-stage NSCLC.^1^, ^2^, ^3^, ^4^

Lymphovascular invasion (LVI) is defined as the presence of neoplastic cells within an arterial, venous, or lymphatic lumen on pathologic examination. LVI is considered a high-risk feature associated with higher rates of nodal involvement and reduced recurrence-free and overall survival.^1^^,^^2^ LVI, along with visceral pleural invasion, a micropapillary predominant pattern within adenocarcinomas, nodal involvement, and older patient age, harbors more aggressive disease biology.^5^ The aim of this large, retrospective analysis was to analyze overall survival and clinical characteristics of segmentectomy vs lobectomy in patients with T1 N0 NSCLC with LVI.

## Patients and Methods

### Data Sources

This retrospective cohort study used the National Cancer Database (NCDB) to identify patients who received a diagnosis of NSCLC between 2004 and 2020. Data were obtained from more than 1500 accredited Commission on Cancer facilities.

### Data Collection and Subject Selection

The initial data file identified 2,023,593 patients with a diagnosis of NSCLC according to the International Classification of Diseases for Oncology, Third Edition (ICD-O-3) guidelines. Patients without LVI on pathology were excluded. Patients with tumors larger than 2 cm, a Charleson-Deyo comorbidity score >0, node-positive disease, missing survival data, or metastasis and patients did not undergo lobectomy or segmental resection were excluded. These exclusion criteria formed a final cohort of 8028 patients with American Joint Committee on Cancer stage I NSCLC with evidence of LVI for statistical testing. These groups were placed in propensity-matched groups, forming a final cohort of 828 patients matched by age, sex, tumor size, and race.

### Statistical Methods

Propensity score matching was used to create 1:1 groups of segmental resection and lobectomy patients matched by age, race, tumor size, and sex. χ^2^ analysis and analysis of variance were used to compare frequencies and denote demographic and clinical differences between the surgical treatment groups. Mean and median survival and survival curves were calculated using pooled log-rank testing and Kaplan-Meier survival analysis. “Crude” and “adjusted” multinomial logistic regression was used to generate odds ratios for surgical treatment and odds of death. The “crude” model contained only the surgical treatment variables, whereas the “adjusted” model included variables for tumor size, median income quartile, and facility type. A Cox proportional hazards model was constructed using only surgical treatment variables to generate hazard ratios. All data were analyzed using SPSS software version 27.0 (IBM Corp). *P* <.05 was used to indicate statistical significance.

### Ethical Considerations

The Participant User Data Files program uses deidentified data and was deemed exempt from Institutional Review Board approval.

## Results

### Demographic and Clinical Characteristics of the Overall Cohort

Of the 8028 patients meeting inclusion criteria, 7612 (94.8%) underwent lobectomy and 416 (5.2%) underwent segmentectomy. Details of baseline demographic characteristics are shown in Supplemental Table 1. Patients who underwent segmentectomy were older, had a later year of diagnosis, and had more frequent treatment in academic or research facilities.

### Tumor Characteristics

The lobectomy group had larger tumor size than the segmental resection group in both unmatched and propensity-matched cohorts. Differences in tumor histologic features were seen between the 2 groups. Patients in the segmental resection group were significantly more likely to have adenocarcinoma compared with patients in the lobectomy group, who had higher rates of squamous cell carcinoma (43.1 vs 35.0; *P* < .001) (Supplemental Table 2).

### Treatment Characteristics

#### Lobectomy

Patients in the unmatched lobectomy group were younger, with a mean age of 67.1 years (*P* < .001), and they more frequently received treatment in nonacademic or research facilities than in academic or research facilities (55.9% vs 44.1%; *P* < .001). Propensity score–matched patients on the basis of age, sex, tumor size, and race are shown in Supplemental Tables 1 and 2.

#### Segmental Resection

Patients in the unmatched segmental resection group were older, with a mean age of 68.9 years (*P* < .001), and they more frequently received treatment in academic or research facilities than in nonacademic or research facilities (50.6% vs 49.4%). They had a shorter mean time to a definitive procedure of the primary site (36.4 days vs 41.3 days; *P* = .027). Propensity score–matched patients on the basis of age, sex, tumor size, and race are shown in Supplemental Tables 1 and 2.

### Overall Survival of Lobectomy vs Segmental Resection

#### Postoperative Outcomes and Survival Analysis: Overall Cohort

The median disease-free survival for patients undergoing lobectomy was 59.6% compared with 58.5% for patients who underwent segmental resection (*P* < .001). There were no significant differences in overall median survival between patients undergoing segmentectomy and those undergoing lobectomy in the unadjusted analysis (74.1 months [95% CI, 72.4-82.6] vs 84.2 months [95% CI, 83.4-86.1], respectively; *P* = .153) (Supplemental Figure). The mean length of stay for patients with lobectomy was longer than in patients with segmentectomy (5.94 days vs 4.56 days, respectively; *P* < .001). There was no significant difference in 30- or 90-day mortality between the 2 groups (Supplemental Table 3).

#### Propensity-Matched Analysis of the Overall Cohort

After 1:1 propensity-score matching, 5-year survival for patients undergoing segmental resection was 58.8% compared with 58.4% for patients undergoing lobectomy (P < 001). There remained no significant differences in overall median survival between patients undergoing segmentectomy and those undergoing lobectomy in the propensity-matched analysis (74.1 months [95% CI, 64.1-86.3] vs 68.7 months [95% CI, 66.1-86.0], respectively; *P* = .334). There were no significant differences in 30- or 90-day mortality between the 2 groups. Multivariate analysis of patients who underwent segmentectomy vs lobectomy showed no difference in hazard between the treatment groups (Cox proportional model for hazard ratio, 1.01; 95% CI, 0.837-1.22; *P* = .922) or odds of death (unadjusted logistic regression, .826; 95% CI, 0.300-0.421; *P* =.178).

## Comment

Lung cancer is the leading cause of cancer-related mortality in men and women, with NSCLC comprising the majority of cases.^1^^,^^2^ Lobectomy has been the standard of care for early-stage node-negative NSCLC. ^6^ Two large, randomized clinical trials determined sublobar resections to be noninferior to lobectomy for T1a N0 NSCLC.^1^^,^^2^ Recurrence was reported in approximately one-third of patients with early-stage disease, thus demonstrating that small tumors may have high-risk features that adversely affect survival.^2^ LVI is a high-risk pathologic feature associated with disease progression, increased recurrence, and decreased survival, but it is not incorporated into treatment recommendations.^8^

After propensity score matching, there was no significant differences in disease-free survival between the treatment groups. This finding is in accordance with studies demonstrating noninferiority of segmental resection to lobectomy in early-stage NSCLC.^1^^,^^2^ Recent large meta-analyses have identified pooled hazard ratios of 1.04 (*P* = .50) and 1.05 (*P* =.550) for segmental resection in NSCLC <2 cm, similar to the hazard ratio of 1.01 identified in the present analysis.^5^^,^^6^ Mathey-Andrews and colleagues^5^ analyzed cT1a-b N0 M0 tumors with evidence of visceral pleural invasion and found no significant difference in survival with lobectomy compared with segmental resection. A significant percentage of their cohort had concomitant LVI and poorly differentiated tumors, a finding suggesting that high-risk features may frequently coexist on histologic analysis. In a subgroup analysis of 163 patients with LVI alone, the investigators found no significant difference in 5-year survival between the treatment groups.^4^ This is similar to the results of the present study, and we were able to build on these findings by analyzing a larger cohort and describing demographic and socioeconomic characteristics for patients with LVI.

Our analysis found that patients undergoing segmental resection were significantly older than those undergoing lobectomy (68.9 years vs 67.1 years, respectively; *P* < .001). Various studies have concluded that surgeons tended to offer segmental resection to patients with stage 1 NSCLC who were older, with poor functional status.^4^ Cao and colleagues^6^ showed no significant difference in survival in patients who could tolerate both lobectomy and segmental resection but significantly worse overall survival for patients undergoing “compromise” segmental resections. Further, there is evidence that sublobar resection may preserve lung function and allow improved postoperative forced expiratory volume in 1 second, forced vital capacity, and diffusing capacity of lung for carbon monoxide compared with lobectomy; however, other studies have produced conflicting results.^7^ Existing studies have shown that length of stay may be a useful surrogate for morbidity after lobectomy in NSCLC.^7^ Our analysis showed segmental resection was associated with a significantly shorter length of stay compared with lobectomy (4.56 days vs 5.94 days; *P* < .01); however, there was no difference in 30-day mortality between the 2 groups. These findings suggest that thoracic surgeons use subtotal resections on older, higher-risk patients and lobectomies in lower-risk patients.

Our results showed that segmental resections were more likely to be performed at academic or research hospitals (50.6% vs 49.4%; *P* = .031). This result correlates with studies analyzing national trends in the quality of segmentectomy for lung cancer and found that the proportion of segmentectomies compared with other resections increased more rapidly at academic programs vs nonacademic programs.^8^ Segmental resection requires thorough intraoperative lymph node sampling to avoid understaging and undertreatment of these patients. Academic or research facilities are better equipped to handle this feature of the procedure and to adhere to quality measures, such as lymph node sampling.^9^

This study has several limitations. The NCDB does not provide cancer-specific mortality data, functional lung status, blood counts, or other comorbidities that factor into treatment decision making and contribute to interpreting outcomes in a clinical setting.^9^ Additionally, the NCDB captures data only from participating institutions, encompassing ∼70% of cancer cases diagnosed nationally.

## References

[1] Saji H., Okada M., Tsuboi M. (2022). Segmentectomy versus lobectomy in small-sized peripheral non-small-cell lung cancer (JCOG0802/WJOG4607L): a multicentre, open-label, phase 3, randomised, controlled, non-inferiority trial. Lancet.

[2] Altorki N., Wang X., Wigle D. (2018). Perioperative mortality and morbidity after sublobar versus lobar resection for early-stage non-small-cell lung cancer: post-hoc analysis of an international, randomized, phase 3 trial (CALGB/Alliance 140503). Lancet Respir Med.

[3] Ginsberg R.J., Rubinstein L.V. (1995). Randomized trial of lobectomy versus limited resection for T1 N0 non-small cell lung cancer. Lung Cancer Study Group. Ann Thorac Surg.

[4] Tosi D., Nosotti M., Bonitta G. (2021). Anatomical segmentectomy versus pulmonary lobectomy for stage I non-small-cell lung cancer: patients selection and outcomes from the European Society of Thoracic Surgeons database analysis. Interact Cardiovasc Thorac Surg.

[5] Mathey-Andrews C., Abruzzo A.R., Venkateswaran S. (2024). Segmentectomy vs lobectomy for early non-small cell lung cancer with visceral pleural invasion. Ann Thorac Surg.

[6] Cao C., Gupta S., Chandrakumar D., Yan T.D. (2014). A critical analysis of segmentectomy versus lobectomy for non-small-cell lung cancer. Eur J Cardiothorac Surg.

[7] Saito H., Nakagawa T., Ito M., Imai K., Ono T., Minamiya Y. (2014). Pulmonary function after lobectomy versus segmentectomy in patients with stage I non-small cell lung cancer. World J Surg.

[8] Rosen J.E., Hancock J.G., Kim A.W. (2014). Predictors of mortality after surgical management of lung cancer in the National Cancer Database. Ann Thorac Surg.

[9] Logan C.D., Jacobs R.C., Feinglass J. (2023). National trends in the quality of segmentectomy for lung cancer. J Thorac Cardiovasc Surg.

