# Supplementary material for: Lobectomy vs Segmentectomy in Stage I Non-Small Cell Lung Cancer With Lymphatic Vascular Invasion
Source: Ann Thorac Surg Short Rep. 2025 Jan 7;3(2):304–7. doi: 10.1016/j.atssr.2024.12.011 (PMC12167564; doi:10.1016/j.atssr.2024.12.011)

Tables

*Table 1: Demographic Characteristics of NSCLC with LVI*

| **Variable** | **Unmatched (N=8028)** | | **p-value** | **Propensity score (1:1) matched (age, sex, race)** | | **p-value** |
| --- | --- | --- | --- | --- | --- | --- |
|  | **Lobectomy**  **(N=7612)** | **Segmental resection**  **(N=416)** |  | **Lobectomy (N=403)** | **Segmental resection**  **(N=403)** |  |
| *Age (mean (SD))* | 67.1 (10.3) | 68.9 (10.9) | <.001 | 68.8 (10.8) | 68.6 (10.8) | 1.00 |
| *Distance travelled (mean(SD))* | 33.7 (120) | 42.1 (114) |  | 21.5 (28.8) | 42.1 (114) | <.001 |
| *Mean Year of diagnosis* | 2012.95 | 2013.02 | .451 | 2011.98 | 2013.02 | <.001 |
| *Sex, n (%)* | | | | | | |
| Male | 3470 (45.6) | 179 (43.0) | .308 | 178 (43.0) | 179 (43.2) | 1.00 |
| Female | 4142 (54.4) | 237 (57.0) |  | 236 (57.0) | 235 (56.8) |  |
| *Race, n (%)* | | | | | | |
| White | 6583 (86.5) | 361 (86.8) | .134 | 335 (87.7) | 359 (86.7) | .202 |
| Black | 627 (8.2) | 26 (6.3) |  | 22 (5.3) | 26 (6.3) |  |
| Other | 402 (5.3) | 29 (7.0) |  | 29 (7.0) | 43 (10.4) |  |
| *Facility Type, n (%)* | | | | | | |
| Community Cancer Program | 282 (3.7) | 17 (4.1) | .031 | 17 (4.1) | 17 (4.1) | .261 |
| Comprehensive Community Cancer Program | 2382 (31.6) | 122 (29.4) |  | 123 (29.7) | 122 (29.5) |  |
| Academic/Research Program | 3325 (44.1) | 210 (50.6) |  | 187 (45.2) | 208 (50.4) |  |
| Integrated Network Cancer Program | 1558 (20.6) | 66 (15.9) |  | 87 (21.0) | 66 (16.0) |  |
| *Insurance Status, n (%)* | | | | | | |
| Uninsured | 124 (1.6) | 4 (1.0) | .194 | 8 (1.9) | 4 (1.0) | .173 |
| Private Insurance | 2434 (32.0) | 129 (31.0) |  | 110 (26.6) | 129 (31.2) |  |
| Medicaid | 360 (4.7) | 14 (3.4) |  | 23 (5.6) | 14 (3.4) |  |
| Medicare | 4501 (59.1) | 264 (63.5) |  | 264 (63.8) | 262 (63.8) |  |
| Other | 85 (1.1) | 1 (0.2) |  | 5 (1.2) | 1 (0.2) |  |
| Insurance status unknown | 105 (1.4) | 4 (1.0) |  | 4 (1.0) | 4 (1.0) |  |
| *Median Income Quartile, n (%)* | | | | | | |
| <$38,000 | 976 (14.7) | 45 (13.2) | .245 | 43 (12.1) | 44 (12.9) | .379 |
| $38,000 - $47,999 | 1380 (20.8) | 65 (19.0) |  | 84 (23.7) | 65 (19.1) |  |
| $48,000 - $62,999 | 1734 (26.1) | 82 (24.0) |  | 91 (25.6) | 82 (24.1) |  |
| ≥$63,00 | 2546 (38.4) | 150 (43.9) |  | 137 (38.6) | 149 (43.8) |  |
| Income data missing | 1050 | |  |  |  |  |

*Table 2: Clinical Characteristics of NSCLC Patients with LVI*

| **Variable** | **Unmatched** | | **p-value** | **Propensity score (1:1) matched** | | **p-value** |
| --- | --- | --- | --- | --- | --- | --- |
|  | **Lobectomy (N=7612)** | **Segmental resection**  **(N=416)** |  | **Lobectomy (N= 403)** | **Segmental resection**  **(N= 403)** |  |
| Tumor Size (Mean (SD) | 34.2 (28.2) | 24.1 (15.7) | <.001 | 24.3 (16.0) | 24.2 (15.7) | .882 |
| *Histology n, (%)* | | | | | | |
| Adenocarcinoma | 2990 (39.3) | 136 (32.7) | <.001 | 163 (39.4) | 136 (32.9) | .077 |
| Squamous Cell Carcinoma | 1310 (17..2) | 61 (14.7) |  | 45 (10.9) | 61 (14.7) |  |
| Other NSCLC | 3312 (43.5) | 219 (52.6) |  | 206 (49.8) | 217 (52.4) |  |
| Definitive Surgical procedure, days from diagnosis (Mean (SD)) | 41.3 (44.1) | 36.4 (44.3) | .027 | 40.5 (57.0) | 36.5 (44.4) | .255 |
| Surgical Inpatient Stay, Days from Surgery (Mean (SD)) | 5.94 (5.80) | 4.56 (3.52) | <.001 | 6.16 (6.35) | 4.56 (3.53) | <.001 |

*Table 3: Overall Survival and 30-day Mortality of NSCLC Patients with LVI*

| **Variable** | **Unmatched** | | **p-value** | **Propensity score (1:1) matched** | | **p-value** |
| --- | --- | --- | --- | --- | --- | --- |
|  | **Lobectomy (N= 7612)** | **Segmental resection**  **(N= 416))** |  | **Lobectomy (N=403)** | **Segmental resection**  **(N= 403)** |  |
| *Median disease-free survival (n,%)* | 84.2 | 74.1 | .153 | 68.7 | 74.1 | .334 |
| *Thirty Day Mortality n (%)* | | | | | | |
| Patient alive, or died more than thirty days after surgery | 7482 (98.3) | 407 (97.8) | .772 | 400 (96.6) | 405 (97.8) | .583 |
| Patient died thirty or fewer days after surgery | 113 (1.5) | 8 (1.9) |  | 12 (2.9) | 18 (1.9) |  |
| Missing data on thirty-day mortality | 17 (0.2) | 1 (0.2) |  | 2 (0.5) | 1 (0.2) |  |
| *Ninety Day Mortality n,(%)* | | | | | | |
| Patient alive, or died more than thirty days after surgery | 7357 (96.7) | 414 (96.9) | .744 | 395 (95.4) | 401 (96.9) | .538 |
| Patient died thirty or fewer days after surgery | 216 (2.8) | 12 (2.9) |  | 17 (4.1) | 12 (2.9) |  |
| Missing data on thirty-day mortality | 39 (0.5) | 1 (0.2) |  | 2 (0.5) | 1 (0.2) |  |

*Figure: Kaplan-Meier Estimate of overall survival for NSCLC patients – propensity matched groups*


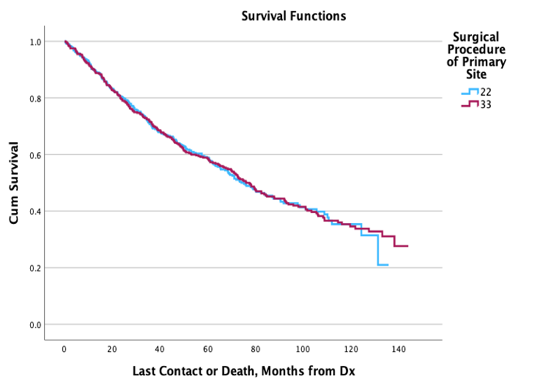

Supplement: Supplemental Material [file mmc1.docx]
